# Single center first year experience and outcomes with Impella 5.5 left ventricular assist device

**DOI:** 10.1186/s13019-022-01871-1

**Published:** 2022-05-23

**Authors:** Joanna R. Rock, Cynthia A. Kos, Anthony Lemaire, Hirohisa Ikegami, Mark J. Russo, Danyaal Moin, Kenneth Dulnuan, Deepa Iyer

**Affiliations:** 1 Rutgers University-Robert Wood Johnson Medical School, New Brunswick, NJ USA; 2 Robert Wood Johnson University Hospital, RWJBarnabas Health, New Brunswick, NJ USA; 3 New Brunswick, USA

**Keywords:** Mechanical circulatory support, Cardiogenic shock, Advanced heart failure, Left ventricular assist devices, Cardiomyopathy, Impella

## Abstract

**Background:**

The Impella 5.5® was approved by the FDA for use for mechanical circulatory support up to 14 days in late 2019 at limited centers in the United States. Our single center’s experience with Impella 5.5® can expand the overall understanding for achieving successful patient outcomes as well as provide support for the expansion of its FDA-approved use.

**Methods:**

This study is an IRB-approved single-center retrospective cohort analysis of hospitalized adult patient characteristics and outcomes in cases where the Impella 5.5® was utilized for mechanical circulatory support.

**Results:**

A total of 26 implanted Impella 5.5® devices were identified in 24 hospitalized patients at our institution from January 2020 to January 2021. The overall survival rate during index hospitalization was 75%. Eleven Impella 5.5® devices were identified in 10 patients with an average device implantation greater than 14 days. Average device implantation for this subgroup was 27 days with a range of 15–80 days. Survival rate for Impella 5.5® use greater than 14 days was 67%. In the entire cohort and subgroup of device implantation > 14 days, evidence of end organ damage improved with Impella 5.5® use. Complications in our cohort and subgroup of device implantation > 14 days were similar to previously reported complication incidence of axillary inserted LVAD devices.

**Conclusions:**

Our institution’s experience with the Impella 5.5® has been strongly positive with favorable outcomes and helps to establish the Impella 5.5® as a viable option for mechanical circulatory support beyond 14 days.

**Supplementary Information:**

The online version contains supplementary material available at 10.1186/s13019-022-01871-1.

## Background

Left ventricular assist devices (LVADs) have evolved since the first implantation by Dr. DeBakey in 1966 and are utilized as a bridge to decision, recovery, or advanced therapies [1, 2]. The overall size of LVADs have decreased throughout the years, but durable LVADs require midline sternotomy or left thoracotomy for left ventricular apical insertion. Minimally invasive LVADs have been developed and successfully used to improve outcomes where emergent and not necessarily durable support is needed [3–5]. The largest of these is a trans-aortic axial flow device, the Impella 5.5® (Abiomed, Danvers MA), which has the capability to deliver a maximum mean of 5.5 L per minute of trans-aortic flow via axillary or central cannulation [6]. This support allows the heart to either recover from cardiogenic shock, cardiotomy, or acute injury and may act as a bridge to recovery, durable device, or transplant in severe cardiomyopathies [7] (Additional file 1: Tables S1, S2).

The Impella 5.5® has several improvements over its predecessors which include increased rigidity, shorter length, and the lack of a pigtail catheter on the ventricular end [8]. These changes allow for easier operator push-ability during implantation and the lack of a pigtail catheter can reduce disruption of the valvular chordae and decrease overall thrombotic risk [8]. Insertion via the axillary artery or aorta along with these modifications facilitates the preservation of patient mobility and can reduce the risk for infection [9–11]. These improvements give the Impella 5.5® the potential to be a durable intra-cardiac LVAD for weeks. Currently, the Impella 5.5® is approved for use for 14 days; however published data for use beyond 14 days is scarce in the United States [6–8, 12].

The following is a summary of our experience with this minimally invasive axial LVAD to expand the overall understanding to achieve successful patient outcomes.

## Methods

### Selection of participants

Participants in this study were determined by a HIPAA-compliant electronic medical record review of hospitalized patients from January 1, 2020- January 1, 2021 who were 18–89 years old and received an Impella 5.5® during their clinical course in Robert Wood University Hospital, New Brunswick, NJ. Indications for use included cardiogenic shock in the setting of acute myocardial infarction or acute systolic heart failure, or for assistance during high-risk percutaneous coronary intervention. Patients were excluded from this study if they did not meet age criteria or did not receive an Impella 5.5® for mechanical circulatory support (Additional file 2: Figs. S1, S2).

### Materials/equipment

The Impella 5.5® with SmartAssist®(Abiomed, Danvers, MA) is a United States Federal Drug Administration (FDA)-approved surgically implanted micro-axial heart pump that delivers up to a maximum mean of 5.5 L of blood per minute from the left ventricle to the aorta. The device itself has been described elsewhere [8]. For insertion, either the right or left axillary artery is isolated via surgical cut-down and anastomosed to a vascular graft through which the pump is ultimately positioned. The Impella 5.5® catheter may also be implanted via direct aortic assess in cases of sternotomy or thoracotomy. Transesophageal echocardiography guides placement regardless of technique. Once in place, the Impella 5.5® requires continuous infusion of heparinized solution via a purge system and requires activated clotting time monitoring. Power and control for the Impella 5.5® is accomplished through an Impella console which displays arterial pressure in the aorta and pressure differences between inflow and outflow outlets.

### Data collection/statistics

Sunrise (Allscripts™) and Muse™ NX (GE Healthcare) electronic health applications were utilized for data collection. Data including demographic, procedural, hemodynamic, laboratory, and outcome values were de-identified and obtained for statistical analysis. Statistical assessment of the data was performed and then expressed as mean ± standard deviation, median (range minimum–maximum), or proportions (percentage) as appropriate. All investigators had access to this study data.

## Results

From January 1, 2020 to January 31, 2021, a total of twenty-six implanted Impella 5.5® minimally invasive axial LVADs were identified in 24 hospitalized patients at our institution. Patients’ ages at implant ranged from 20 to 81 years old (Mean of 51 ± 31 years) with 92% identified as male. Average length of Impella 5.5® use was 15 days (± 15) with the longest duration of implantation being 80 days (Table 1).

**Table 1:** Patient characteristics for the entire cohort

Characteristic	All Patients	Patients with Impella > 14 days
	n = 26*	n = 11**
Median age (range)—yr	66 (20–81)	58 (20–72)
Sex—no. (%)		
Male	22 (92%)	9(90%)
Female	2 (8%)	1 (10%)
Ethnicity—no. (%)		
Asian	3 (13%)	2 (20%)
Hispanic	1 (4%)	1 (10%)
Non-hispanic black	2 (8%)	1 (10%)
Non-hispanic white	18 (75%)	6 (60%)
BMI (range)—avg no	29 (18–43)	28 (18–36)
Etiology of Shock- no. (%)		
Acute MI	11 (46%)	5 (50%)
HFrEF	8 (33%)	5 (50%)
Cardiotomy	5 (21%)	0
Functional Status—no. (%)		
Bedbound	18 (69%)	7 (70%)
OOB/Walking	8 (31%)	3 (30%)
LVEF—avg %	21%	16%
Comorbidities—no. (%)		
Atrial Fibrillation	1 (4%)	0
CAD (h/o PCI/CABG)	10 (42%)	5 (50%)
CKD	4 (17%)	1 (10%)
COPD	1 (4%)	0
Diabetes Mellitus	5 (21%)	3 (30%)
Hyperlipidemia	11 (46%)	4 (40%)
Hypertension	14 (58%)	6 (60%)
PVD	3 (13%)	0
Stroke	2 (8%)	1 (10%)
Tobacco use, current	4 (17%)	1 (10%)
End-organ function—avg (± SD)		
Hemoglobin—g/dL	11.0 (± 2.4)	10.7 (± 2.5)
Platelets—u/µL	200 (± 127)	220 (± 184)
Lactate—mmol/L	4.6 (± 4.0)	5.0 (± 3.5)
Creatinine—mg/dL	2.1 (± 0.9)	1.7 (± 0.6)
BUN- mg/dL	39 (± 24)	40 (± 25)
AST/ALT—u/L	457(± 794) / 352(± 694)	559(± 1027) / 635(± 1043)
Sodium	137 (± 7)	134 (± 9)
Hemodynamics—avg (± SD)		
RA—mmHg	12 (± 6)	12 (± 8)
PA, systolic—mmHg	48 (± 13)	49 (± 16)
PA, diastolic—mmHg	23 (± 6)	23 (± 8)
PCWP—mmHg	26 (± 7)	25 (± 9)
PAPi	2.2 (± 0.7)	4 (± 4)
PA VO2—%	54.2 (± 11.9)	50.9 (± 11.3)
Hemodynamic support—avg (± SD)		
Vasopressors	2 (± 1)	2 (± 1)
Intubated—no. (%)	15 (58%)	5 (50%)
Implant site—no. (%)		
Direct	4 (15%)	0
Left axillary artery	2 (8%)	1 (10%)
Right axillary artery	20 (77%)	9 (1%)

*Two patients underwent re-implantation

**One patient underwent re-implantation

The predominant indication for Impella 5.5® support was cardiogenic shock secondary to acute myocardial infarction with 11 instances (46% total). Of those, 10 cases were with ST segment elevation myocardial infarction and 1 case with non-ST segment elevation myocardial infarction in which use was for high-risk percutaneous coronary intervention support. The second most predominant indication was for low output congestive systolic heart failure (33%), followed by cardiotomy (21%). The majority of implants were via right axillary artery regardless of indication (77%). Those implants that were via left axillary artery (7%) were for Impella 5.5® exchange after device malfunction and those that were via direct aortic access (15%) were during cardiotomy.

The overall mortality during index hospitalization was 25%. All deaths were in the setting of acute myocardial infarction or high risk percutaneous coronary intervention. Of the patients in the cohort, 75% survived. Out of those who survived, 83% had recovery of native heart function, 6% underwent cardiac transplantation, and 11% underwent durable LVAD placement.

Prior to Impella 5.5® implantation, 58% required mechanical ventilatory support of which 40% were able to be extubated after placement. All patients off mechanical ventilatory support prior to Impella 5.5® placement were able to be extubated post implant. Average time to extubation was 5 (± 5) days post-Impella 5.5® implantation.

At the time of Impella 5.5® placement, most patients were bed-bound (70%). Of the bed-bound patients after implant, 39% were walking or mobile to bedside chair and post-explant, 50% were walking or mobile to chair. Additional information can be found in Supplementary Information-Additional file 2.

Invasive hemodynamics were documented on 18 of the patients. Of those patients, CVP improved from an average of 12 mmHg (± 6) prior to Impella 5.5® to 8 mmHg (± 5) at explant. Mean PA pressure improved from 33 mmHg (± 7) to 23 mmHg (± 10). Pulmonary artery pulsatility index (PAPi) improved from 2.2 (± 0.7) prior to implant to 5.8 (± 4.9) at explant (Table 2).

**Table 2:** General invasive hemodynamic pressures for the entire cohort

	Baseline	POD#1	POD#5	Explant
CVP/RA	12 (± 6)	10 (± 4)	9 (± 5)	8 (± 5)
PA, systolic	48 (± 13)	38 (± 7)	41 (± 9)	37 (± 15)
PA, diastolic	23 (± 6)	18 (± 8)	20 (± 6)	20 (± 12)
PA, mean	33 (± 7)	25 (± 5)	28 (± 8)	23 (± 10)
PCWP	26 (± 7)	18 (± 6)	18 (± 6)	14 (± 7)
PAPi	2.2 (± 0.7)	2.6 (± 1.5)	2.2 (± 0.9)	5.8 (± 4.9)
PA VO2	54.2 (± 11.9)	68.8 (10.5)	66.9 (± 9.7)	68.8 (± 10.3)
Hemodynamic support—no. (%)				
Vasopressors	2 (± 1)	2 (± 1)	1 (± 1)	1 (± 1)

**Fig. 1:**
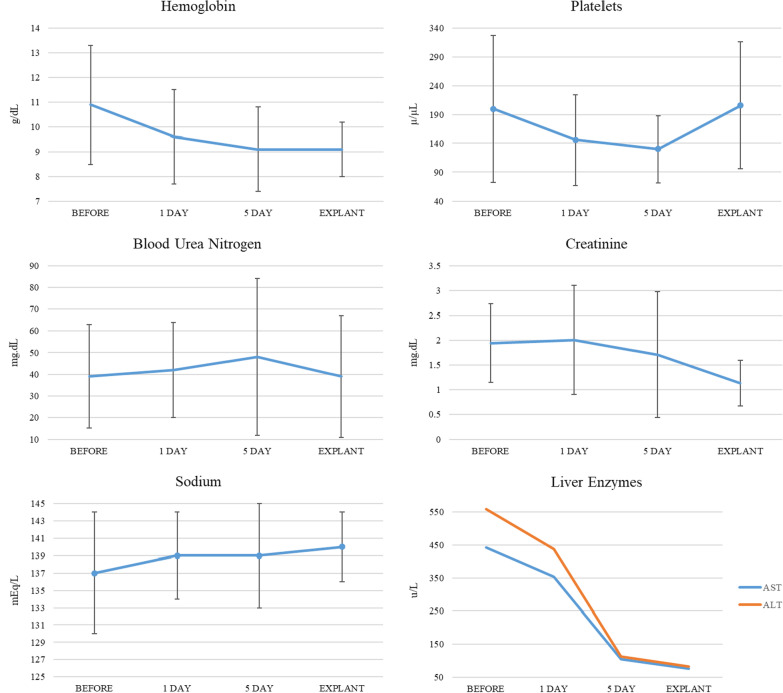
General laboratory values for entire cohort. Trends in laboratory values show improvement in kidney and liver function with Impella 5.5 use. Hemoglobin and platelet both show an initial decrease after implantation with stabilization at time of explant

On average, laboratory values showed an improvement in oxygen exchange and end organ function. Of note, mixed venous saturation improved from 54.2% (± 11.8) prior to implantation to 68.8% (± 10.3) at explant. Also, lactate down trended from 4.6 (± 4.0) mmol/L pre-implantation to 1.5 (± 0.6) mmol/L at explant. Using creatinine as a surrogate, kidney function improved after impella 5.5 use. Prior to implantation average creatinine was 1.94 (± 0.79)mg/dL then after initial increase to 2.0 (± 1.10) mg/dL one day after implantation, creatinine improved to 1.13 (± 0.46) mg/dL at explant. Liver enzymes, aspartate transaminase and alanine transaminase (AST and ALT), saw an initial rise from 442 (± 917) units/L and 503 (± 937) units/L to 354 (± 772) units/L and 548 (± 1362) units/L respectively one day post implant. AST and ALT levels then decreased to 76 (± 86) units/L and 53 (± 55) units/L at time of explant. Hemoglobin overall remained stable with a slight downtrend during implantation from an average of 10.9 (± 2.4) g/dL at implant to 9.6 (± 1.9) g/dL at one day, to ultimately 9.1 (± 1.1) g/dL at explant. Platelets decreased initially with an average of 200,000 (± 127,000) µL at time of implant to 146,000 (± 78,000) µL at day one post implant and 130,000 (± 58,000) µL day five of implant. At the time of explant platelets had improved to 206,000 (± 110,000) µL (Table 3) (Fig. 1).

**Table 3:** General lab values for the entire cohort

	Baseline	POD#1	POD#5	Explant
Hgb—g/dL	10.9 (± 2.4)	9.6 (± 1.9)	9.1 (± 1.7)	9.1 (± 1.1)
Platelets—u/µL	200 (± 127)	146 (± 78)	130 (± 58)	206 (± 110)
Lactate—mmol/L	4.6 (± 4.0)	2.2 (± 1.8)	1.1 (± 0.4)	1.4 (± 0.6)
Creatinine—mg/dL	1.94 (± 0.79)	2.0 (± 1.10)	1.71(± 1.27)	1.13(± 0.46)
BUN—mg/dL	39 (± 24)	42 (± 22)	48 (± 36)	39 (± 28)
AST/ALT—units/L	442/503 (± 917/ ± 937)	354/548 (± 772/ ± 1362)	104/83 (± 139/ ± 78)	76/53 (± 86/ ± 55)

Twenty-three patients received echocardiograms before and after Impella 5.5 implantation during their hospitalization. Improvement in ventricular function was defined as an increase in left ventricular ejection fraction greater than 5% and an improvement in qualitative right ventricular assessment grading of mild, moderate, or severe. Ventricular assessment was taken from the official reports that were finalized during their hospital stay. Ventricular function improved for 39% of patients for the left ventricle and 44% for the right ventricle. Of those with improved left ventricular function, the ejection fraction improved an average of 14% (± 7.7%) (Table 4).

**Table 4:** General echocardiographic values for the entire cohort

	Percentage of patients with improved LVEF	Average LVEF improvement of those who improved	Percentage of patients with improved RV function
All patients	36% (n = 10)	14% (± 7.7%)	40%
Acute myocardial Infarction	40% (n = 4)	15% (± 6.1%)	44%
Cardiomyopathy	33% (n = 3)	17% (± 9.4%)	60%
Post-cardiotomy	40% (n = 2)	4% (± 4.1%)	0%
Assistance for high-risk PCI	0	0	0

The most common patient complications were gastrointestinal bleeding (15%), axillary hematoma at insertion site (15%), stroke (8%), development of heparin induced thrombocytopenia (8%), and infection at insertion site (4%) (Table 5). All of the axillary hematomas and infections required operative excision, exploration, and washout. One of the gastrointestinal bleedings was severe requiring massive transfusion protocol in which the patient developed acute pulmonary edema, hypoxia, and pulseless electrical activity requiring resuscitation. Ultimately that patient was made comfort measures by the family. There were two instances of device malfunction requiring exchange (8%). One malfunction was a software complication in which the Impella 5.5® device abruptly shut off requiring patient hemodynamic resuscitation and exchange. In that case, the patient survived and was ultimately discharged from the hospital. The other device malfunction was the development of a physical crack in the purge cassette of the Impella 5.5® purge delivery system which had to be temporarily sutured together until a new Impella 5.5® could be exchanged to the left axillary artery. In that case, the patient was successfully hemodynamically stabilized however family ultimately decided to withdraw care instead of having a durable LVAD implanted.

**Table 5:** Complications of entire cohort vs. patients with implantation > 14 days

Complication	Entire Cohort n = 26	Greater than 14 days n = 11
Overall complication rate	12 (46%)	7 (73%)
Axillary infection	1 (4%)	1 (9%)
Axillary hematoma	4 (15%)	2 (18%)
Device malfunction	2 (8%)	2 (18%)
Gastrointestinal bleed	4 (15%)	2 (18%)
Stroke	2 (8%)	1 (9%)
HIT positive	2 (8%)	1 (9%)
New ESRD with HD	2 (8%)	1 (9%)

Eleven LVADs in ten patients were identified with an average device implantation for greater than 14 days. Average device implantation for this subgroup was 27 days with a range of 15–80 days. Two of these patients had to undergo an Impella 5.5® re-implantation via the left axillary artery due to device malfunction. Of the subgroup with Impella 5.5® use for greater than 14 days, the median age was 58 with range 20–72 years old. The indication for implantation included cardiogenic shock due to ST-segment elevation myocardial infarction (55%), low output congestive heart failure (36%), and assistance for high-risk percutaneous coronary intervention for non-ST segment elevation myocardial infarction (9%). Complications and rates are listed in Table 5. Survival rate for Impella 5.5® use greater than 14 days was 67%. Of those who survived, 50% had native heart recovery, 17% had durable device implantation, and 33% cardiac transplant. All of the patients who expired were in the setting of cardiogenic shock after acute myocardial infarction. Of the patients who expired, 50% of them were made comfort care after hemodynamic stabilization and goals of care discussions.

**Fig. 2:**
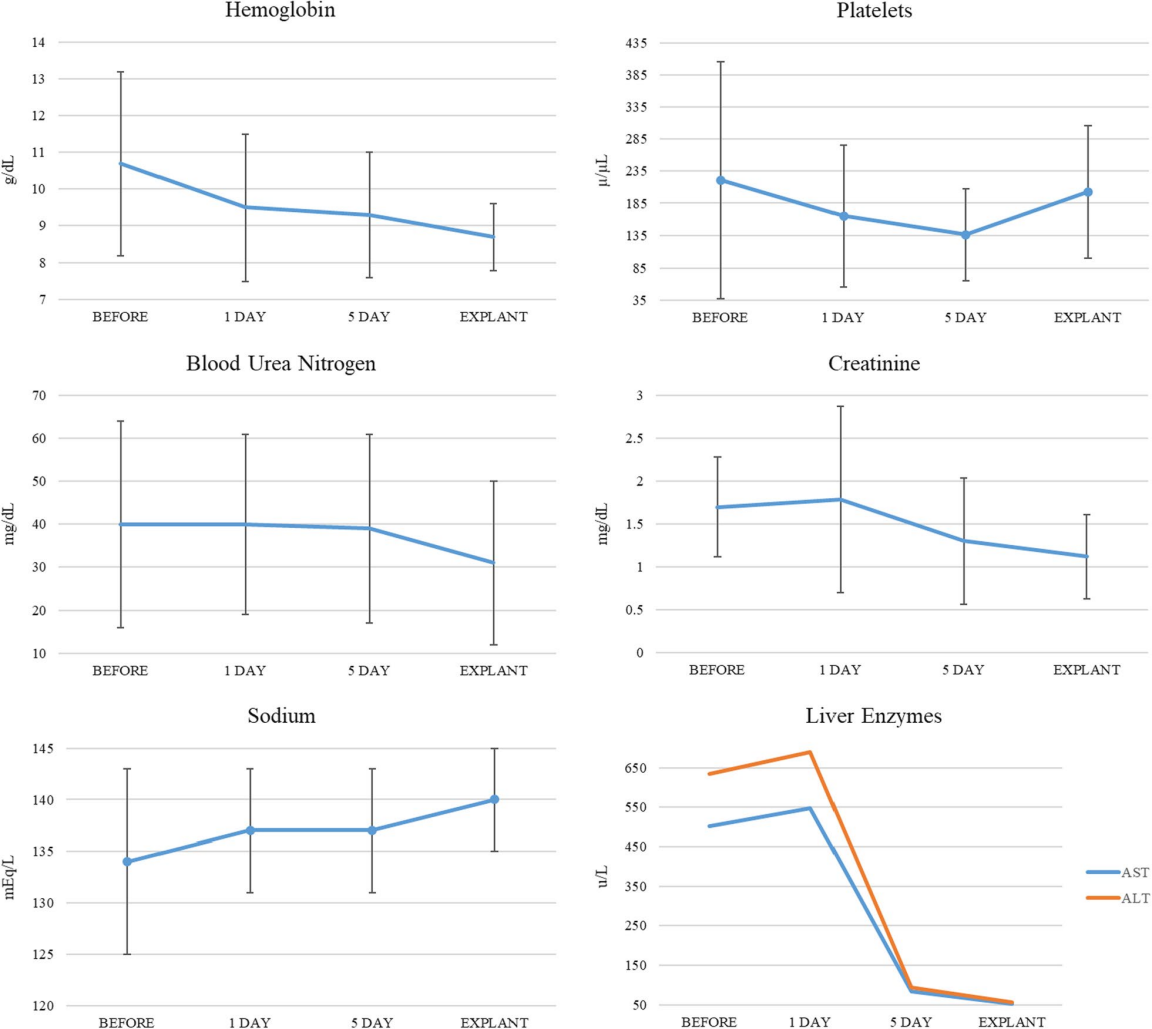
General laboratory values for patients with implantation > 14 days. Trends in laboratory values show improvement in kidney and liver function with Impella 5.5 use for > 14 days. Liver enzymes first worsened in the 24 h after implant after stabilization and improvement with time. Hemoglobin and platelet both show an initial decrease after implantation with stabilization at time of explant

Laboratory trends in those patients who had the Impella 5.5® for greater than 14 days were similar to the entire group. Data is listed in Supplementary Information-Additional file 1. Overall complications were higher than the comprehensive cohort at 73% over the 46% for the entire cohort (Fig. 2).

## Discussion

Our center utilized the Impella 5.5® in treatment of cardiogenic shock from myocardial infarction and systolic heart failure, support for high-risk percutaneous coronary intervention and cardiotomy. Despite treatment, cardiogenic shock can lead to inadequate cardiac output causing end organ failure with a high mortality rate of approximately 40% [13]. With use of the Impella 5.5® for cardiogenic shock, our center had an 75% survival to hospital discharge with native heart recovery, durable LVAD implantation, or cardiac transplant. Our center’s overall improvement in survival to hospital discharge confirms the Impella 5.5® is a valid choice for the treatment of cardiogenic shock.

Furthermore, our center’s experience highlights the Impella 5.5®’s ability to unload the increased pressures of the heart to reverse organ damage. Hemodynamic monitoring showed improvement in biventricular cardiac filling pressures which correlated to improvement seen on echocardiography.

The improved organ perfusion was confirmed with laboratory monitoring before, during, and after use of Impella 5.5 ®. Lactate, BUN/creatinine, and liver enzyme studies all decreased with Impella 5.5® use. The initial worsening of creatinine and liver enzyme studies are expected as both biomarkers can lag upwards of 3–4 days after the reversal of cardiogenic shock [14, 15]. Both hemoglobin and platelet levels decreased during Impella 5.5® implantation however no patient experienced an iatrogenic sudden decrease in these levels suggesting minimal cellular shearing by the device. In addition, platelet counts stabilized at time of explant to near pre-implant levels suggesting that the left-ventricular unloading had a positive effect on reversing thrombocytopenia secondary to congestive hepatopathy and splenomegaly [14, 16].

Gastrointestinal bleeding and axillary hematoma were the most prevalent complications in our cohort at 15% each and in the subgroup of patients with implantation > 14 days at 18% each. Our gastrointestinal bleeding incidence in both groups fall within the reported range of 13–30% [17]. Published data on bleeding events at the Impella insertion site suggest a rate of 12–30% for femoral artery-inserted Impella devices (2.5 and CP) and 11–15% for axillary artery-inserted Impella devices (5.0/LD) [10, 18–20].

Hemolysis rates for axillary Impella devices (5.0/LD) have been previously reported at 6.5% which was an improvement from their femoral artery-inserted Impella device counterparts which had a reported hemolysis rate of 15% [10, 18–20]. Our cohort did not have any major hemolysis events and even saw a reversal of hemolysis in one patient after the patient was upgraded from the Impella CP to the Impella 5.5®.

Most of the patients showed an improvement in mobility while implanted with the Impella 5.5® and no patient in our cohort had a limb complication from the device. Previously published prevalence of limb complications from femoral-inserted Impella devices (2.5 and CP) was 9% [19, 20]. The re-location of the device to the axilla allows for participation in physical therapy and may also decrease infection rates as our experience had only one case of infection at insertion site (4%) whereas previous rates of infection were in range of 12–18% when the groin was used [21].

Of the deaths at our institution, all patients were in cardiogenic shock after acute ST segment or non-ST segment myocardial infarctions. Our survival rate of 50% for cardiogenic shock after acute myocardial infarction is slightly better than the published estimated in-hospital survival rate of 40% which was prior to the advent of LVADs in the 1990’s [22]. In our single case of Impella 5.5 ® use for high-risk PCI, the patient initially presented with non-ST segment elevation myocardial infarction and refused surgical revascularization therefore undergoing high-risk PCI and was unable to be weaned off the Impella 5.5® post-procedure. That patient was not be a candidate for LVAD or cardiac transplant at our institution and an outside institution and comfort-directed care was pursued by the patient and family.

In the United States, current Impella 5.5® use has been approved for 14 days. In Europe, the approval is extended to 30 days. Our center has successfully used the Impella 5.5® for periods longer than 14 days in range of 15 to 80 days. For the patients who were implanted for greater than 14 days, the survival rate to hospital discharge was 64%. Improved hemodynamics and organ perfusion similar to the entire cohort was confirmed with invasive hemodynamic and laboratory. Our center’s experience supports the hypothesis that the Impella 5.5® is a durable alternative for left ventricular mechanical circulatory support beyond 14 days.

While our findings are promising for the consideration of the Impella 5.5® in acute cardiogenic shock protocols, our study did have several limitations as single-center, retrospective review. Our sample bias may limit extrapolation of our experience to the general population in that our cohort was overwhelmingly male gender (85.7% male versus 14.3% female) and identified as non-Hispanic white (75%). This may represent gender bias and is consistent with other reported studies evaluating patient selection for mechanical circulatory support. Those studies suggest that women are more likely to be diagnosed later with end-stage heart failure, transferred at a more critical state, and have overall worse outcomes after LVAD implantation than their male counterparts [1, 23, 24]. In addition, our institution did not utilize the Impella 5.5® for any female who had an acute myocardial infarction. The estimated proportion of females out of patients with acute myocardial infarction in the state of New Jersey is 42% [25]. This can be assumed to also be representative of our institution as we are a large regional tertiary care center in New Jersey. This gender bias may be a result of under-recognition of heart failure and acute myocardial infarction in our female community as their presentation often differs from their male counterparts and is independently associated with a higher in-hospital mortality [26]. This experience brings about the opportunity for our institution to investigate educational opportunities for the correction of this gender gap in care in our community and our own practice. Second, our implanted patient population was majorly non-Hispanic white (75%) with underrepresentation of non-Hispanic African Americans (8%) and Hispanic populations (4%). Published population studies have shown the incidence of heart failure is more evenly distributed among different ethnic groups with proposed incidences being 39% for non-Hispanic white, 29% for non-Hispanic African American, and 23% Hispanic [27]. Our study highlights a disparity among racial groups which may be reflective of proposed socioeconomic inequalities in the treatment of heart failure.

Our study as a retrospective cohort introduces bias as the variables were collected during a comprehensive chart review. Data collection was limited as missing variables were often encountered during chart review due to variation in medical care team documentation. This can be alleviated in future prospective studies with the creation of a treatment documentation algorithm thereby having all variables accounted for in the electronic medical record.

Future prospective studies evaluating patient outcomes after establishing the patient’s baseline characteristics can create a more comprehensive assessment of the Impella 5.5’s® role in the treatment of cardiogenic shock. In addition, studies which are more representative of the racial and gender distribution of our general population will be useful to see if special considerations are needed in their treatment course. Nonetheless, our institution’s experience with the Impella 5.5® has been strongly positive with favorable outcomes and helps to establish the Impella 5.5® as a viable option for mechanical circulatory support beyond 14 days.

## Clinical perspective

The summary of our experience with Impella 5.5® can expand the overall understanding of use in order to achieve successful patient outcomes as well as provide support for the expansion of its FDA-approved use. In addition our experience highlights a bias in mechanical circulatory support against the female sex and minority groups which is under-recognized and not fully understood. We are hopeful that our experience can help improve outcomes with other centers in their comprehensive care for cardiogenic shock.

## Supplementary Information

**Additional file 1: Table S1.** Invasive Hemodynamic pressures for patients with implant >14 days. **Table S2.** Lab Values for patients implanted >14 days.

**Additional file 2: Fig. S1.** Functional Status for the entire cohort. Functional status improved with Impella 5.5 placement as axillary location allows for greater participation with physical therapy and for ambulation. **Fig. S2.** Respiratory Status for the Entire Cohort. A majority of patients were able to be taken off a respirator during Impella 5.5 implantation as hemodynamics and cardiopulmonary status improved with the Impella 5.5.

## Data Availability

The datasets generated and/or analyzed during the current study are not publicly available due to constraints of IRB approval but are available from the corresponding author on reasonable request.

## Abbreviations

LVADs: Left ventricular assist devices
PAPi: Pulmonary artery pulsatility index
AST: Aspartate transaminase
ALT: Alanine transaminase
BUN: Blood urea nitrogen
