# Supplementary material for: Single center first year experience and outcomes with Impella 5.5 left ventricular assist device
Source: J Cardiothorac Surg. 2022 May 23;17:124. doi: 10.1186/s13019-022-01871-1 (PMC9128113; doi:10.1186/s13019-022-01871-1)
Supplement: Supplementary file 1 — Additional file 1: Table S1. Invasive Hemodynamic pressures for patients with implant >14 days. Table S2. Lab Values for patients implanted >14 days. [file 13019_2022_1871_MOESM1_ESM.docx]

Supplemental Figures/Tables

Table 1. Invasive Hemodynamic Pressures for Patients with Implant >14 days

|  | Baseline | | POD#1 | POD#5 | Explant |
| --- | --- | --- | --- | --- | --- |
| CVP/RA | 12 (±8) | | 9 (±5) | 9 (±6) | 8 (±5) |
| PA, systolic  PA, diastolic  PA, mean | 49 (±16)  23 (±8)  34 (±8) | | 39 (±9)  16 (±7)  26 (±6) | 44 (±8)  18 (±6)  29 (±7) | 36 (±15)  16 (±8)  23 (±10) |
| PCWP | 25 (±9) | | 20(±8) | 16 (±7) | 14 (±7) |
| PAPi | 2.4 (± 1.3) | | 3.0 (±1.9) | 4.6 (±4.4) | 3.7 (±3.4) |
| PA VO2 | | 50.9 (±11.3) | 68.4 (±9.5) | 68.3 (±10.8) | 66.2 (±7.0) |
| Hemodynamic support – no. (%)  Vasopressors | | 2 (±1) | 2 (±1) | 1 (±1) | 1 (±1) |

Table 2. Lab Values for Patients Implanted >14 days

|  | Baseline | | POD#1 | POD#5 | Explant |
| --- | --- | --- | --- | --- | --- |
| Hgb – g/dl | 10.7 (±2.5) | | 9.5 (±2.0) | 9.3 (±1.7) | 8.7 (±0.9) |
| Platelets – u/µL | 221 (±184) | | 165 (±110) | 136 (±71) | 203 (±103) |
| Lactate – mmol/L | 5.0 (±3.5) | | 2.2 (±2.4) | 1.2 (±0.5) | 1.4 (±0.4) |
| Creatinine –mg/dL | 1.70 (±0.58) | | 1.79 (±1.09) | 1.30(±0.74) | 1.12(±0.49) |
| BUN- mg/dL | | 40 (±24) | 40 (±21) | 39(±22) | 31 (±19) |
| AST/ALT – u/L | | 559/635  (±1026/±1043) | 438/689 (±871/±1541) | 113/93  (±157/±87) | 82/57  (±89/±57) |

Supplemental Graph 1. Functional Status for the Entire Cohort.

Functional status improved with Impella 5.5 placement as axillary location allows for greater participation with physical therapy and for ambulation.

Supplemental Graph 2. Respiratory Status for the Entire Cohort.

A majority of patients were able to be taken off a respirator during Impella 5.5 implantation as hemodynamics and cardiopulmonary status improved with the Impella 5.5.
