# Supplementary figures and images for: Single center first year experience and outcomes with Impella 5.5 left ventricular assist device
Source: J Cardiothorac Surg. 2022 May 23;17:124. doi: 10.1186/s13019-022-01871-1 (PMC9128113; doi:10.1186/s13019-022-01871-1)

Supplemental Graph 1

## Functional Status

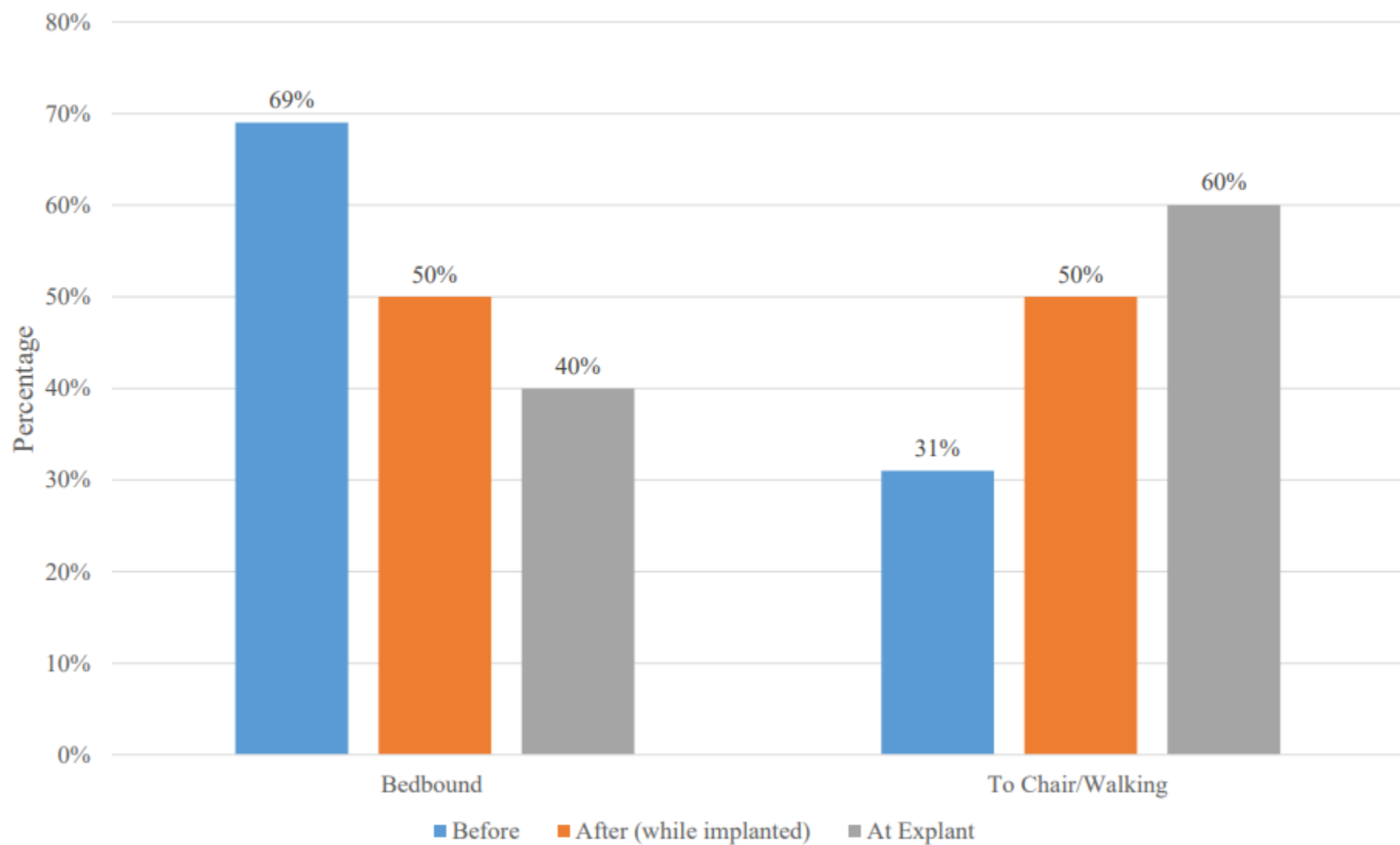

Supplemental Graph 1

# Respiratory Status

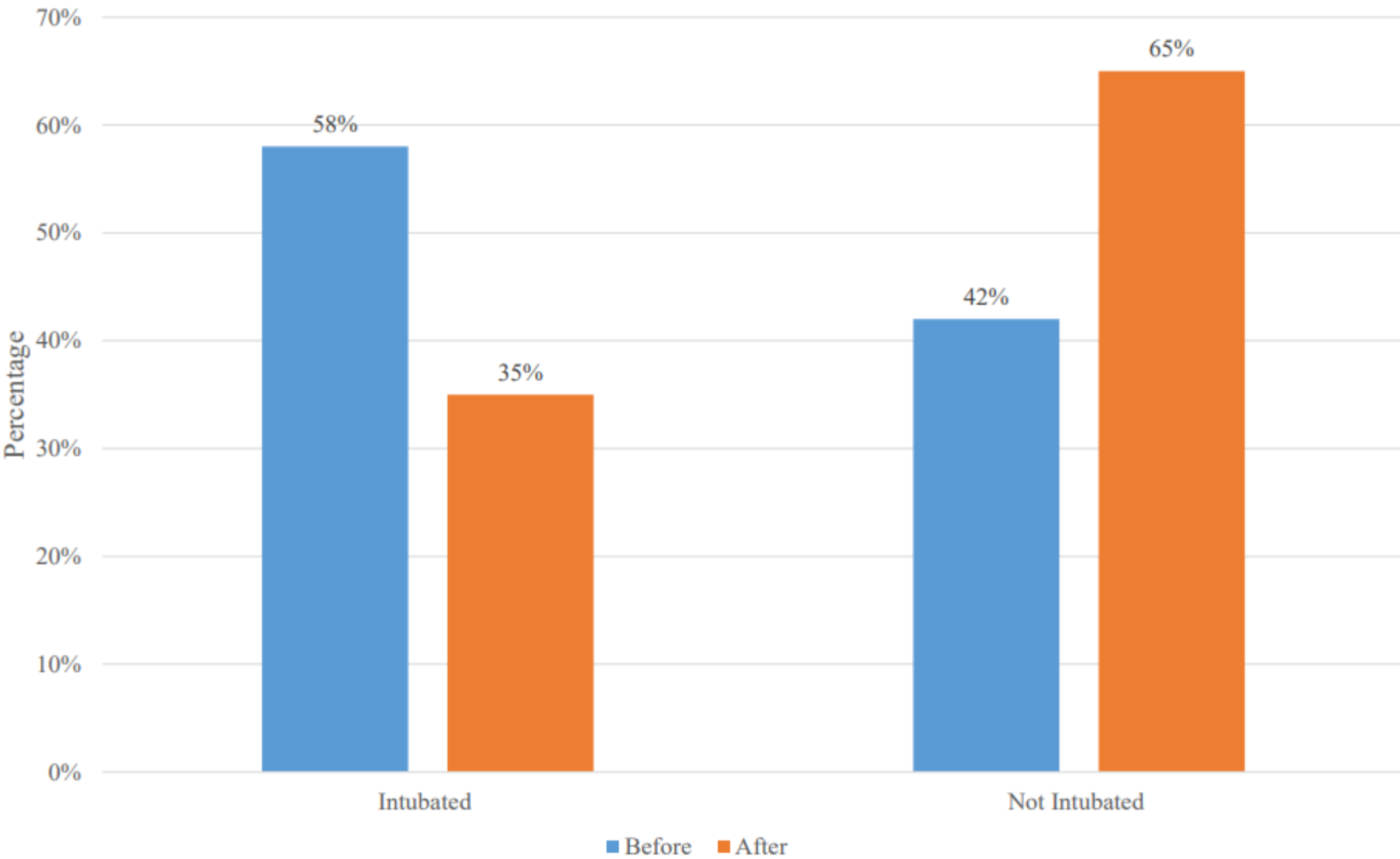

Supplement: Supplementary file 2 — Additional file 2: Fig. S1. Functional Status for the entire cohort. Functional status improved with Impella 5.5 placement as axillary location allows for greater participation with physical therapy and for ambulation. Fig. S2. Respiratory Status for the Entire Cohort. A majority of patients were able to be taken off a respirator during Impella 5.5 implantation as hemodynamics and cardiopulmonary status improved with the Impella 5.5. [file 13019_2022_1871_MOESM2_ESM.pdf]
